# Heat therapy preserves myofibre size and SERCA‐mediated Ca^2+^ uptake in the mouse soleus after tenotomy surgery

**DOI:** 10.14814/phy2.70385

**Published:** 2025-05-22

**Authors:** Michael K. Barfoot, Jessica L. Braun, Phillip J. Wallace, Bianca M. Marcella, Ryan W. Baranowski, Rebecca E. K. MacPherson, Stephen S. Cheung, Val A. Fajardo

**Affiliations:** ^1^ Department of Kinesiology Brock University St. Catharines Ontario Canada; ^2^ Centre for Bone and Muscle Health Brock University St. Catharines Ontario Canada; ^3^ Department of Health Sciences Brock University St. Catharines Ontario Canada

**Keywords:** calpain, HSP70, muscle unloading, SERCA

## Abstract

Heat therapy (HT) has been shown to induce physiological adaptations in muscle, including a reduction in the severity of muscle atrophy resulting from unloading. The muscle atrophy caused by unloading can be partially attributed to the dysregulation of Ca^2+^ in the muscle cell, which can activate calpain‐mediated proteolysis. The sarco(endo)plasmic reticulum Ca^2+^‐ATPase (SERCA) is a primary regulator of Ca^2+^ in muscle, and SERCA dysfunction has been repeatedly demonstrated in various models of muscle unloading. Heat shock protein 70 (HSP70) is a heat‐inducible chaperone protein that binds to SERCA and protects against its dysfunction. While previous research has shown HT to upregulate HSP70 in rodent muscle, even in the unloaded state, the effects of HT on SERCA function in rodent skeletal muscle under these conditions remain unknown. Here, we characterized the effects of 4 weeks of HT on soleus muscle size, HSP70 expression, SERCA function, and maximal calpain activity in male C57BL/6J mice subjected to muscle unloading through tenotomy. Four weeks of HT preserved the cross‐sectional area of soleus myofibres following tenotomy, while also upregulating HSP70, maintaining SERCA‐mediated Ca^2+^ uptake, and reducing maximal calpain activity. Therefore, our research offers new insights into the advantages of HT for muscle health and physiology.

## 1 INTRODUCTION

Skeletal muscle constitutes 40%–50% of total body mass and is vital for daily functions such as mobility and metabolism (Frontera & Ochala, 2015; Wolfe, 2006). The significance of skeletal muscle is evident in conditions such as sarcopenia—age‐related losses in muscle mass and strength—that repeatedly serve as a significant independent predictor of all‐cause mortality (Benz et al., 2024; Brown et al., 2016; Zhang et al., 2018; Zhou et al., 2023). Muscle size is dynamic, and it is well established that regular resistance training leads to increases in skeletal muscle size and strength, while muscle unloading or disuse—common in situations such as aging, spaceflight, bed rest, or surgical tenotomy—results in muscle atrophy and weakness (Baranowski et al., 2023; Braun et al., 2021; Fajardo et al., 2017). In light of the importance of skeletal muscle, programs aimed at increasing muscle gains and minimizing muscle loss during periods of training and inactivitywould greatly support muscle health and overall well‐being.

In recent years, heat therapy (HT) has emerged as a potential therapeutic aid in the rehabilitation of muscle injuries and in managing conditions related to chronic pain and increased stiffness (Kim et al., 2020; Nadler et al., 2004). Furthermore, additional research has shown that HT can enhance muscle size both in response to training and in the absence of it. It also improves muscle size recovery after unloading or disuse while reducing the muscle atrophy associated with these conditions, establishing HT as a potential strategy for preserving muscle mass (for review, see ref. (Kim et al., 2020)). For example, in rats, a single session of HT reduced the level of soleus muscle atrophy (by approximately 30%) resulting from 8 days of hindlimb suspension (Naito et al., 2000). This finding was associated with a significant increase in heat shock protein 70 (HSP70), a heat‐inducible chaperone protein that can enhance cellular resilience against stressors such as oxidative stress (Ilievska et al., 2018; Naito et al., 2000).

HSP70 is also a well‐known regulator and protector of the sarco(endo)plasmic reticulum Ca^2+^‐ATPase (SERCA), which catalyzes the active transport of Ca^2+^ into the sarcoplasmic reticulum. In turn, SERCA triggers muscle relaxation and sustains resting levels of free intracellular Ca^2+^ at approximately 100 nM (Tupling, 2004). Previous studies have shown that HSP70 can physically interact with SERCA to preserve its function in conditions of heightened oxidative stress (Fu & Tupling, 2009; Tupling et al., 2004). In the context of muscle disuse and unloading, impairments to SERCA function have been found across a variety of models including hindlimb suspension (Marais et al., 2025), tenotomy (Fajardo et al., 2017), spaceflight (Braun et al., 2021), and denervation (Dufresne et al., 2016). These impairments to SERCA function can result in the accumulation of cytosolic Ca^2+^, which activates proteolytic enzymes such as calpain, leading to muscle atrophy and weakness (Fennel et al., 2022; Gao et al., 2018). Overall, this suggests that a potential mechanism through which HT preserves soleus muscle size during unloading may involve upregulating HSP70, which then maintains SERCA function and decreases calpain‐mediated proteolysis. To our knowledge, this has yet to be tested. Therefore, as a first step to addressing this knowledge gap, we aimed to characterize the effects of HT on soleus muscle size, HSP70 content, SERCA function, and calpain activity in response to unloading caused by tenotomy.

## 2 MATERIALS AND METHODS

### 2.1 Animals

All animal protocols were approved by the Brock University Animal Care Committee (AUP #21‐04‐03). Male C57BL/6J mice (3–6 months old) were ordered from Jackson Laboratories. The mice were housed in standard 12‐h light:12‐h dark cycles and were provided with standard rodent chow (2014 Teklad Global, Inotiv) and water. All mice were allowed to acclimate to the animal care facilities at Brock University for 1 week prior to undergoing tenotomy surgery. After tenotomy surgery (described below), mice were randomly divided into two groups: HT or control (CON), *n* = 16 per group.

### 2.2 Tenotomy

Following the acclimation period, all mice underwent the tenotomy surgery while anesthetized with 2% vaporized isoflurane as previously described (Baranowski et al., 2022; Fajardo et al., 2017). Briefly, on one leg, the tendons of the gastrocnemius and soleus were exposed and transected, and the wound was closed with a silk braided suture. The contralateral leg served as a sham control, whereby a small incision was made; however, the soleus and gastrocnemius tendons were left intact. Silk braided suture was also used to close the wound on the sham control leg. Following surgery, the mice were provided with oral Metacam treatments daily for 5 days. Following this 5‐day period, the mice were subject to their respective treatment (i.e., HT or CON) for 4 weeks every 72 h.

### 2.3 Heat and control therapy

The heating protocol was adapted from a previous study (Von Schulze et al., 2021) and consisted of passive heating to a target core temperature and a 20‐min treatment at the target temperature. Each mouse performed the protocol at the same time of day to account for circadian fluctuations in core temperature. The treatments were repeated every 72 h to limit any potential heat adaptation for a total of nine exposures. Furthermore, HT every 72 h has been shown to induce a robust increase in HSP70 in the rat heart (Ilievska et al., 2018). For all exposures, mice were anesthetized with isoflurane and transferred to a nose cone on a metallic bead bath (74300–706; Cornelius, OR, USA) with continued anesthesia. Upon transfer, 0.5 mL of saline was injected subcutaneously for hydration. A rectal thermocouple (RET‐4, Physitemp, NJ, USA) was inserted and taped to the tail and bead bath to prevent slippage and potential tissue damage. Temperature was recorded using a data logger (TC‐2000 Type‐T Thermocouple Meter, Sable Systems International, NV, USA) connected to an online data acquisition software (Labchart V7.3.8, AD Instruments, CO, USA) sampled at 1 Hz. The bead bath was set at 38.5°C in CON and ~44°C in HT. During heating, a red housing dome was placed over the mice to create a microclimate. Both air temperature (°C) in the dome and bead bath temperature (°C) under the mouse was recorded using a thermocouple. In CON, mice were passively heated to a core temperature range between 37.0°C and 38.0°C, where the housing dome was removed and the 20‐min treatment was started when core temperature reached 37.0°C. In HT, mice were passively heated to a core temperature range between 40.5°C and 41.5°C, where the 20‐min treatment started when core temperature reached 40.5°C. To slow the rise in core temperature, the housing dome was removed when core temperature reached 40.0°C. A paw pulse oximeter (MouseSTAT Jr., Kent Scientific Corporation, CT, USA) was placed on the right hind paw to monitor heart rate throughout the treatment period as a safety precaution. When heart rate rose above 500 b∙min,^−1^ the isoflurane was reduced by 0.5%. If heart rate would not decrease with up to 3 decreases in isoflurane, the trial was terminated. Following the 20‐min treatment, the mice were provided with another 0.5 mL of saline via subcutaneous injection, had their anesthesia removed, and were then transferred to a recovery cage. The mice were sacrificed 72 h after their final (i.e., 9th) treatment.

### 2.4 Tissue collection

Following the 4 weeks of HT or CON treatment, animals were sacrificed via exsanguination under general anesthesia (vaporized isoflurane) 3 days after their last respective treatment. Both soleus muscles (sham and tenotomy) were dissected and stored at −80°C. A subset of the soleus muscles was embedded in Optimal Cutting Temperature (OCT) compound (ThermoFisher Scientific; 6,769,006; USA) and cooled with liquid N_2_ prior to being stored at −80°C for histological assessment. Another subset of soleus muscles was homogenized in a buffer consisting of 5 mM HEPES, 250 mM sucrose, 0.2 mM PMSF, and 0.2% NaN3 at a pH of 7.5 for the purposes of calcium uptake and western blot analysis. Additionally, another subset was homogenized in RIPA buffer to conduct calpain assays.

### 2.5 Calcium uptake

Ca^2+^ uptake assays were performed in homogenized soleus samples using an Indo‐1 based spectrofluorimetric assay previously described by our group (Geromella et al., 2023). Indo‐1 is a ratioable Ca^2+^ dye that has a single excitation wavelength of 355 nm and two optimal emission wavelengths: 405 nm in the Ca^2+^‐bound state and 485 nm in the Ca^2+^‐free state. The ratio of Ca^2+^‐bound to Ca^2+^‐free Indo‐1 (Indo‐1_405/485nm_) provides an indication of free [Ca^2+^]_i_ (Braun et al., 2021; Geromella et al., 2023). Briefly, muscle homogenate was added to reaction buffer (200 mM KCl, 20 mM HEPES, 10 mM NaN_3_, 5 μM TPEN, 15 mM MgCl_2_, 5 mM oxalate (pH 7.0)) containing Indo‐1 (4 μM final concentration; 57,180, Sigma‐Aldrich, St. Louis, MO, USA). Samples were then plated in duplicate, to which ATP (10 mM final concentration) was added to initiate Ca^2+^ uptake. Indo‐1_405/485nm_ was measured kinetically using a Molecular Devices M2 plate reader (Molecular Devices, San Jose, CA, USA) upon excitation at 355 nm at 37°C for a period of 500 s. Area‐under‐the‐curve (AUC) analyses provide a measure of the amount of Ca^2+^ transported by SERCA over time. Rates of Ca^2+^ uptake were also measured within the first 88 s of the uptake protocol by obtaining the slope from linear regression analyses. This time point was chosen as it represents the 1st and fastest phase of Ca^2+^ uptake with an *R* ^2^ = 0.97 ± 0.02. All rates of Ca^2+^ uptake were normalized to total protein content (in g) measured with a bicinchoninic acid (BCA) assay.

### 2.6 H&E staining

Myofibre size was determined via Hematoxylin and Eosin (H&E) staining of samples from mice from the control and HT groups that were previously embedded in OCT. The soleus muscles were sliced into 10 μm sections using a ThermoFisher Scientific HM525NX Cryostat (956,640; UK) and then placed onto microscope slides treated with VECTABOND (SP‐1800‐7, VectorLabs, Newark, CA, USA). All slides were then imaged using a BioTek Cytation 5 Multimode Plate Reader (8,040,036; Winooski, VT, USA). Three images from each slide were randomly acquired at 10× magnification. The cross‐sectional area of 30 myofibres from each soleus (*n* = 10 fibers measured per image) was analyzed using imageJ software (NIH).

### 2.7 Calpain assay

A commercialized calpain assay (QIA120; Millipore Sigma) was used to determine maximal calpain activity in the soleus muscles. Tissue was freshly homogenized in RIPA lysis buffer (10:1 dilution), and 50 μL of sample was loaded in duplicate in either 100 μL of activator buffer or 100 μL of inhibitor buffer in a 96‐well plate as per the manufacturer's instructions. Then, 50 μL of diluted substrate was added in the dark into each well, and the plate was incubated for 15 min. The plate was then read at an excitation of 370 nm and emission of 450 nm. Maximal calpain activity was then calculated by subtracting the OD value obtained from the inhibitor wells from those obtained in the activator wells. Data were presented as relative fluorescence units and normalized to total protein (in mg), with each n representing the average obtained from one soleus sample run in duplicate.

### 2.8 Western blotting

Western blotting was conducted to determine the content of the following proteins: SERCA1, SERCA2, HSP70, and nitrotyrosine. Antibodies for SERCA1 (MA3‐911; ThermoFisher Scientific) and SERCA2 (MA3‐919; ThermoFisher Scientific) were obtained from ThermoFisher Scientific (Rockford, IL, USA). The antibodies for HSP70 (4872S; Cell Signaling Technology) and nitrotyrosine (9691S; Cell Signaling Technology) were obtained from Cell Signaling Technology (Danvers, MA, USA). Western blots were performed using BioRad TGX precast gels (4%–15% gradient) (Bio‐Rad Laboratories, USA), a transblot turbo (Turbo setting) and polyvinylidene difluoride (PVDF) membranes. Prior to incubating membranes with the primary antibodies, all membranes were blocked with BioRad EveryBlot blocking buffer (12,010,020, BioRad Laboratories) for 15 min at room temperature. Primary antibodies were diluted in either 5% non‐fat milk and were applied to the membranes overnight at 4°C. Following the overnight incubation, membranes were washed 3 times with Tris‐buffered saline tween (TBST) for 5 min each, after which the secondary antibodies (anti‐mouse or anti‐rabbit) and milk were applied to the membranes for 1 h at room temperature. Membranes were then washed 3× in TBST prior to detection with either Immobilon® ECL Ultra Western HRP Substrate (WBKLS0500; Burlington, MA, USA) using a BioRad ChemiDoc Imager. A Ponceau stain was then done to determine total protein content and to normalize obtained values from the analyses.

### 2.9 Statistical analysis

Comparisons were made using either a Student's *t*‐test or a two‐way ANOVA that assessed the main effects of tenotomy, HT, and their potential interaction. When necessary, a Tukey's post‐hoc test was used. Statistical significance was set to *p* < 0.05. Data are presented as means ± SD.

## 3 RESULTS

To characterize the effects of HT on soleus muscle size, HSP70 expression, SERCA function, and maximal calpain activity in the unloaded state, male C57BL/6J mice were subjected to unilateral tenotomy surgery to unload one (left hindlimb) soleus muscle. The contralateral leg served as a sham control. Five days post‐surgery, mice were divided into two groups, HT or CON. The HT group received 20 min exposure to a heated bead bath at a target core temperature of 40.5°C–41.5°C, every 72 h for 4 weeks, whereas the CON group received 20 min exposure to the bead bath with a target core temperature of 37.0°C–38.0°C. A total of 274 of 288 trials were completed, with 11 trials (~8%) stopped in the HT group due to high core temperature or heart rates, where durations in these trials ranged between 7 and 15 min. Three trials (~2%) were stopped in CON due to high heart rate, where trials ranged between 4 and 14 min. Across all exposures, core temperature was significantly higher in the HT group (~41.0°C ± 0.1°C) compared to CON (~37.3°C ± 0.1°C) (Figure S1).

**FIGURE 1:**
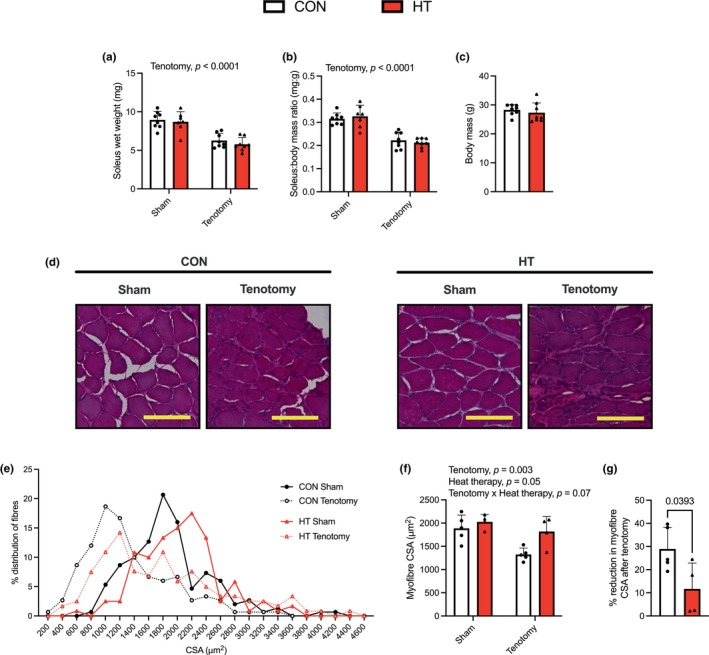
Heat therapy (HT) preserves soleus myofibre cross‐sectional area (CSA) after tenotomy. (a) Absolute soleus muscle mass (mg). (b) Soleus muscle mass (mg) relative to body mass (g). (c) Body mass (g). (d) Representative H&E images. Scale bars are set to 200 μm. (e) Frequency distribution graphs plotted by CSA (μm^2^). (f) Average myofibre CSA. For each *n*, 3 images were randomly taken with a total of 30 myofibres measured. (g) % reduction in myofibre CSA (Tny vs. sham) in the CON and HT groups. For a, b, and f, data was analyzed with a two‐way plot ANOVA (*n* = 3–5 males per group). For c and g, data was analyzed with a Student's *t*‐test. CON, control; Tny, tenotomy. All values are presented as mean ± SD, and exact *p*‐values are reported.

After the 4 weeks, all mice were euthanized and the soleus muscles were collected. Soleus muscle mass, both absolute and relative to body mass, was significantly lower with tenotomy in both HT and CON groups (Figure 1a,b). There were no differences in body mass between groups at the end of the study period (Figure 1c). Collectively, the measurements of soleus wet weight show that both the HT and CON groups experienced comparable levels of soleus muscle atrophy following tenotomy surgery. However, it is worth noting that since tenotomy surgery severs the soleus and gastrocnemius tendons, we were unable to isolate the soleus muscle from tendon to tendon, which is required for reliable and consistent measures of soleus muscle mass. Thus, the assessment of soleus myofibre cross‐sectional area (CSA) may be a more reliable and accurate outcome measure. In this respect, histological assessments using H&E stains revealed a preservation of myofibre size after HT (Figure 1d–f). That is, while tenotomy resulted in a leftward shift in the CSA frequency distribution plot (Figure 1e) and a significant reduction in average myofibre CSA (Figure 1f) in both CON and HT groups, heat exposure induced opposing effects, including a rightward shift in the CSA frequency distribution plot and a significant increase in average myofibre CSA. Moreover, when we compared the % reduction in myofibre size after tenotomy between HT and CON groups, we found that the CON group experienced a significantly larger % decline in myofibre size (−28%) versus the HT group (−11%) (Figure 1g), which altogether demonstrates that HT attenuated the decline in soleus myofibre size.

**FIGURE 2:**
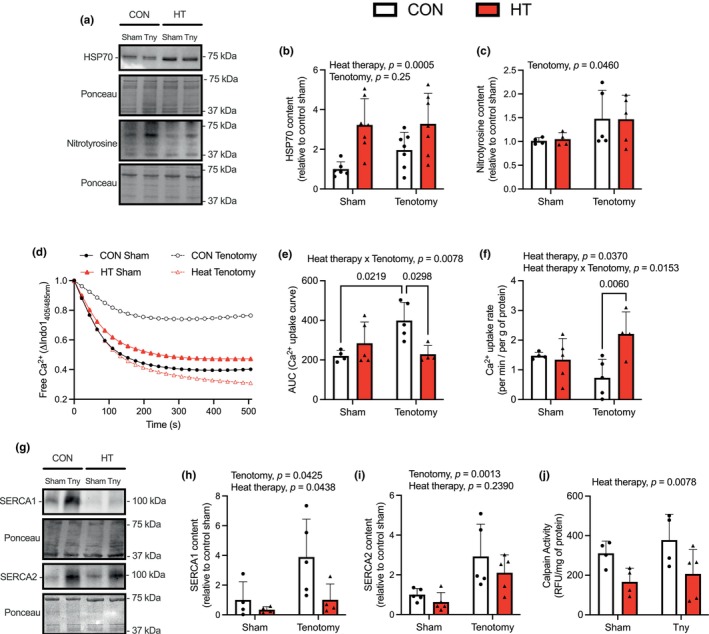
Heat therapy (HT) upregulates HSP70 and maintains SERCA function in the tenotomized soleus. (a) Representative western blot images and semi‐quantitative analysis of HSP70 (b, *n* = 6–7 per group) and protein tyrosine nitration (c, *n* = 4–5 per group). (d) Representative Ca^2+^ uptake curves across all experimental groups. Data are presented as the change in Indo‐1_405/485nm_, which is indicative of the amount of free intracellular Ca^2+^. (e) AUC analyses for the Ca^2+^ uptake curve. (f) Normalized rates of Ca^2+^ uptake with the slopes obtained via linear regression analysis within the first 88 s of Ca^2+^ uptake, which was then divided by protein content. For d–f, *n* = 4–5 per group. (g) Representative western blot images and semi‐quantitative analysis of SERCA1 (h, *n* = 4–5 per group) and SERCA2 (i, *n* = 5 per group). (j) Maximal calpain activity in the soleus homogenates, *n* = 4–5 per group. For b, c; e, f; and h‐j; data were analyzed with a two‐way ANOVA and a Tukey's post‐hoc test. All values are presented as mean ± SD, and exact *p*‐values are reported.

We then conducted western blot analysis to confirm that HT would upregulate HSP70 content in the soleus. Consistent with previously published findings after only one session of HT (Naito et al., 2000), our results with 4 weeks of HT show that HSP70 content was higher in both sham and tenotomized soleus muscles (Figure 2a,b). We consider the upregulation of HSP70 after HT to be beneficial, particularly since tenotomy was shown to induce oxidative stress, as indicated by significantly higher levels of protein nitrotyrosine (Figure 2a,c).

Next, we investigated the effects of tenotomy and HT on SERCA function in the soleus muscle (Figure 2d–f). The area under the curve during our Ca^2+^ uptake experiments—indicating the total amount of Ca^2+^ in the cytosol during the uptake period—was significantly elevated in the CON tenotomy group but decreased to sham levels in the tenotomized HT group (Figure 2e). Moreover, normalized rates of Ca^2+^ uptake were also significantly slower in the tenotomized soleus from the CON group compared with the tenotomized soleus from the HT group (Figure 2f). Regarding SERCA isoform content, we found that HT lowered both SERCA1 and SERCA2 isoforms; albeit, the main effect of HT was not significant in the latter (Figure 2g,h). Furthermore, a main effect of tenotomy for both isoforms indicates that tenotomy surgery leads to greater levels of SERCA protein in the soleus (Figure 2g,h). Lastly, we measured calpain activity in the soleus muscles from sham and tenotomy groups and found that HT significantly lowered maximal calpain activity in soleus muscles from both conditions (Figure 2j).

## 4 DISCUSSION

Our study characterized the effects of 4 weeks of HT on murine soleus muscle size, HSP70 content, SERCA function, and maximal calpain activity in an unloaded state caused by surgical tenotomy. Our results indicate that male C57BL/6J mice undergoing tenotomy surgery and 4 weeks of HT exhibited preserved soleus myofibre CSA, increased HSP70 levels, maintained SERCA function, and reduced maximal calpain activity.

The elevation of HSP70 with HT observed in this study was expected and aligns well with findings from a prior study conducted on rats, which demonstrated that a single session of heat therapy can upregulate HSP70 in muscle tissue (Naito et al., 2000). We consider the increase in HSP70 to be advantageous in the context of the unloaded soleus, where we observed indications of heightened oxidative stress associated with elevated levels of protein tyrosine nitration. In relation to SERCA, the upregulation of HSP70 is important because this Ca^2+^ pump contains several residues, including tyrosine, as well as cysteine and lysine that are highly susceptible to free radical attack. When oxidized, these residues lead to a significant reduction in SERCA pump activity and Ca^2+^ uptake (Viner et al., 1996, 1997, 1999). To preserve SERCA function, chaperone proteins, namely HSP70, can physically interact with SERCA, protecting it from oxidative and nitrosative modification and impairment (Fu & Tupling, 2009; Gehrig et al., 2012; Tupling et al., 2004). In accordance with this, our Ca^2+^ uptake experiments indicate that SERCA function was preserved in the HT group following tenotomy. However, determining whether the preservation of SERCA function is mainly due to the upregulation of HSP70 with HT requires further investigation that systematically examines the role of HSP70 in maintaining SERCA function in response to soleus tenotomy.

Moreover, improvements to SERCA‐mediated Ca^2+^ uptake were found despite there being significant reductions in SERCA content after HT. Western blot analysis of SERCA1 and SERCA2, the dominant isoforms found in skeletal muscle (Tupling, 2004), shows that SERCA1 content was significantly lower in the HT group compared to CON. A similar pattern was observed with SERCA2; however, the main effect of HT only had a *p*‐value of 0.2390. Additionally, a significant main effect of tenotomy on both SERCA isoforms was observed, where tenotomy increased the content of SERCA1 and SERCA2. Altogether, these data suggest that despite lowering SERCA content, HT enhanced SERCA‐mediated Ca^2+^ uptake following tenotomy, potentially by increasing HSP70. Moreover, we acknowledge that variations in SR Ca^2+^ leak through Ca^2+^ channels (i.e., lowered Ca^2+^ leak from ryanodine receptors with HT) may also contribute to the differences observed in our Ca^2+^ uptake experiments since our protocol represents the net balance between Ca^2+^ uptake and leak in and out of the SR, respectively. Thus, this should be investigated further with future studies.

Finally, our results also show that HT significantly reduced maximal calpain activity in both the sham and tenotomized soleus. In a previous study using pulmonary microvascular endothelial cells, heat exposure attenuated lipopolysaccharide‐induced apoptosis and proteolysis partly by lowering calpain expression and activity (Liu et al., 2016). While we anticipate that HT would have similar effects on calpain expression and activity in the murine soleus, constraints on sample volume prevented us from conducting western blot analysis on total and activated (autolyzed) calpain content. In addition to limits in sample volume, our study is also limited by the fact that we utilized only male mice. Future studies should investigate whether HT preserves muscle mass, upregulates HSP70, maintains SERCA function, and reduces maximal calpain activity in female mice to assess any potential influence of biological sex.

In conclusion, the present study contributes to an expanding body of literature highlighting the therapeutic benefits of HT in muscle (Kim et al., 2020). Others have shown that HT can alleviate muscle atrophy caused by unloading, with various cellular mechanisms proposed, including but not limited to the promotion of mitochondrial function, an increase in protein synthesis, and a reduction in protein breakdown (Fennel et al., 2022; Goto et al., 2004; Hafen et al., 2018, 2019; Hunt et al., 2019; Kim et al., 2020; Yoshihara et al., 2015). The results from our study offer novel insights by demonstrating that HT can preserve SERCA function and reduce calpain activity in response to muscle unloading caused by tenotomy. However, future studies using other models of unloading and disuse would help determine if our findings are generalizable. Nonetheless, although this study did not establish the exact cellular mechanisms behind these effects, our results provide further evidence for the benefits of HT on muscle health and physiology.

## AUTHOR CONTRIBUTIONS

**Michael K. Barfoot:** Writing and drafting the manuscript, data collection, data interpretation, and reviewing and revising the manuscript. **Jessica L. Braun:** Data collection, data interpretation, reviewing, and revising the manuscript. **Phillip J. Wallace:** Data collection, data interpretation, reviewing, and revising the manuscript. **Bianca M. Marcella:** Data collection, data interpretation, reviewing, and revising the manuscript. **Ryan W. Baranowski:** Data collection, data interpretation, reviewing, and revising the manuscript. **Rebecca E. K. MacPherson:** Concept design, provision of reagents and resources, acquiring funding, reviewing and revising the manuscript. **Stephen S. Cheung:** Concept design, provision of reagents and resources, acquiring funding, reviewing, and revising the manuscript. **Val A. Fajardo:** Concept design, provision of reagents and resources, acquiring funding, supervision, writing, and drafting the manuscript.

## FUNDING INFORMATION

This work was supported by an NSERC Discovery Grant to VAF (2019–05833) and SSC (2018–04077). Michael K. Barfoot is supported by an Ontario Graduate Scholarship and was supported by an NSERC USRA. Jessica L. Braun is supported by a CIHR Doctoral Award. Phillip J. Wallace was supported by an Ontario Graduate Scholarship. Bianca M. Marcella is supported by a CIHR CGS‐M. Val A. Fajardo is supported by a Canada Research Chair Tier II Award in Tissue Plasticity and Remodeling throughout the Lifespan.

## CONFLICT OF INTEREST STATEMENT

The authors declare no conflicts of interest.

## Supporting information


Figure S1.

## Data Availability

All data supporting the results presented in this manuscript can be made available upon reasonable request.

## References

[phy270385-bib-0001] Baranowski, R. W. , Braun, J. L. , Hockey, B. L. , Yumol, J. L. , Geromella, M. S. , Watson, C. J. F. , Kurgan, N. , Messner, H. N. , Whitley, K. C. , MacNeil, A. J. , Gauquelin‐Koch, G. , Bertile, F. , Gittings, W. , Vandenboom, R. , Ward, W. E. , & Fajardo, V. A. (2023). Toward countering muscle and bone loss with spaceflight: GSK3 as a potential target. iScience, 26, 107047. 37360691 10.1016/j.isci.2023.107047 PMC10285634

[phy270385-bib-0002] Baranowski, R. W. , Braun, J. L. , Vandenboom, R. , & Fajardo, V. A. (2022). Neurogranin inhibits calcineurin in murine soleus muscle: Effects of heterozygous knockdown on muscle adaptations to tenotomy and fatigue resistance. Biochemical and Biophysical Research Communications, 623, 89–95. 35878428 10.1016/j.bbrc.2022.07.062

[phy270385-bib-0003] Benz, E. , Pinel, A. , Guillet, C. , Capel, F. , Pereira, B. , De Antonio, M. , Pouget, M. , Cruz‐Jentoft, A. J. , Eglseer, D. , Topinkova, E. , Barazzoni, R. , Rivadeneira, F. , Ikram, M. A. , Steur, M. , Voortman, T. , Schoufour, J. D. , Weijs, P. J. M. , & Boirie, Y. (2024). Sarcopenia and sarcopenic obesity and mortality among older people. JAMA Network Open, 7, e243604. 38526491 10.1001/jamanetworkopen.2024.3604 PMC10964118

[phy270385-bib-0004] Braun, J. L. , Geromella, M. S. , Hamstra, S. I. , Messner, H. N. , & Fajardo, V. A. (2021). Characterizing SERCA function in murine skeletal muscles after 35–37 days of spaceflight. International Journal of Molecular Sciences, 22, 11764. 34769190 10.3390/ijms222111764 PMC8584217

[phy270385-bib-0005] Brown, J. C. , Harhay, M. O. , & Harhay, M. N. (2016). Sarcopenia and mortality among a population‐based sample of community‐dwelling older adults. Journal of Cachexia, Sarcopenia and Muscle, 7, 290–298. 27239410 10.1002/jcsm.12073 PMC4864252

[phy270385-bib-0006] Dufresne, S. S. , Dumont, N. A. , Boulanger‐Piette, A. , Fajardo, V. A. , Gamu, D. , Kake‐Guena, S. A. , David, R. O. , Bouchard, P. , Lavergne, E. , Penninger, J. M. , Pape, P. C. , Tupling, A. R. , & Frenette, J. (2016). Muscle RANK is a key regulator of calcium storage, SERCA activity, and function of fast‐twitch skeletal muscles. American Journal of Physiology‐Cell Physiology, 310(8), C663–C672. 26825123 10.1152/ajpcell.00285.2015 PMC4835920

[phy270385-bib-0007] Fajardo, V. A. , Rietze, B. A. , Chambers, P. J. , Bellissimo, C. , Bombardier, E. , Quadrilatero, J. , & Tupling, A. R. (2017). Effects of sarcolipin deletion on skeletal muscle adaptive responses to functional overload and unload. American Journal of Physiology. Cell Physiology, 313, C154–C161. 28592414 10.1152/ajpcell.00291.2016

[phy270385-bib-0008] Fennel, Z. J. , Amorim, F. T. , Deyhle, M. R. , Hafen, P. S. , & Mermier, C. M. (2022). The heat shock connection: Skeletal muscle hypertrophy and atrophy. American Journal of Physiology. Regulatory, Integrative and Comparative Physiology, 323, R133–R148. 35536704 10.1152/ajpregu.00048.2022

[phy270385-bib-0009] Frontera, W. R. , & Ochala, J. (2015). Skeletal muscle: A brief review of structure and function. Calcified Tissue International, 96, 183–195. 25294644 10.1007/s00223-014-9915-y

[phy270385-bib-0010] Fu, M. H. , & Tupling, A. R. (2009). Protective effects of Hsp70 on the structure and function of SERCA2a expressed in HEK‐293 cells during heat stress. American Journal of Physiology. Heart and Circulatory Physiology, 296, H1175–H1183. 19252085 10.1152/ajpheart.01276.2008

[phy270385-bib-0011] Gao, Y. , Arfat, Y. , Wang, H. , & Goswami, N. (2018). Muscle atrophy induced by mechanical unloading: Mechanisms and potential countermeasures. Frontiers in Physiology, 9, 235. 29615929 10.3389/fphys.2018.00235 PMC5869217

[phy270385-bib-0012] Gehrig, S. M. , van der Poel, C. , Sayer, T. A. , Schertzer, J. D. , Henstridge, D. C. , Church, J. E. , Lamon, S. , Russell, A. P. , Davies, K. E. , Febbraio, M. A. , & Lynch, G. S. (2012). Hsp72 preserves muscle function and slows progression of severe muscular dystrophy. Nature, 484, 394–398. 22495301 10.1038/nature10980

[phy270385-bib-0013] Geromella, M. S. , Braun, J. L. , & Fajardo, V. A. (2023). Measuring SERCA‐mediated calcium uptake in mouse muscle homogenates. STAR Protocols, 4, 101987. 36602905 10.1016/j.xpro.2022.101987 PMC9826970

[phy270385-bib-0014] Goto, K. , Honda, M. , Kobayashi, T. , Uehara, K. , Kojima, A. , Akema, T. , Sugiura, T. , Yamada, S. , Ohira, Y. , & Yoshioka, T. (2004). Heat stress facilitates the recovery of atrophied soleus muscle in rat. The Japanese Journal of Physiology, 54, 285–293. 15541206 10.2170/jjphysiol.54.285

[phy270385-bib-0015] Hafen, P. S. , Abbott, K. , Bowden, J. , Lopiano, R. , Hancock, C. R. , & Hyldahl, R. D. (2019). Daily heat treatment maintains mitochondrial function and attenuates atrophy in human skeletal muscle subjected to immobilization. Journal of Applied Physiology (Bethesda, MD: 1985), 127(1), 47–57. 10.1152/japplphysiol.01098.2018 31046520

[phy270385-bib-0016] Hafen, P. S. , Preece, C. N. , Sorensen, J. R. , Hancock, C. R. , & Hyldahl, R. D. (2018). Repeated exposure to heat stress induces mitochondrial adaptation in human skeletal muscle. Journal of Applied Physiology (Bethesda, MD: 1985), 125(5), 1447–1455. 10.1152/japplphysiol.00383.2018 30024339

[phy270385-bib-0017] Hunt, A. P. , Minett, G. M. , Gibson, O. R. , Kerr, G. K. , & Stewart, I. B. (2019). Could heat therapy Be an effective treatment for Alzheimer's and Parkinson's diseases? A narrative review. Frontiers in Physiology, 10, 1556. 31998141 10.3389/fphys.2019.01556 PMC6965159

[phy270385-bib-0018] Ilievska, G. , Dinevska‐Kjovkarovska, S. , & Miova, B. (2018). Effect of single and repeated heat stress on chemical signals of heat shock response cascade in the rat's heart. Cell Stress & Chaperones, 23, 561–570. 29178005 10.1007/s12192-017-0863-0 PMC6045549

[phy270385-bib-0019] Kim, K. , Monroe, J. C. , Gavin, T. P. , & Roseguini, B. T. (2020). Skeletal muscle adaptations to heat therapy. Journal of Applied Physiology (Bethesda, MD: 1985), 128, 1635–1642. 32352340 10.1152/japplphysiol.00061.2020 PMC7311689

[phy270385-bib-0020] Liu, Z. F. , Zheng, D. , Fan, G. C. , Peng, T. , & Su, L. (2016). Heat stress prevents lipopolysaccharide‐induced apoptosis in pulmonary microvascular endothelial cells by blocking calpain/p38 MAPK signalling. Apoptosis, 21, 896–904. 27325431 10.1007/s10495-016-1263-0 PMC5069199

[phy270385-bib-0021] Marais, A. A. T. , Baranowski, R. W. , Braun, J. L. , Hockey, B. L. , & Fajardo, V. A. (2025). Targeting GSK3 to attenuate spaceflight‐induced SERCA dysfunction: Lessons from hindlimb‐suspended mice. Biochimica et Biophysica Acta, Molecular Basis of Disease, 1871(3), 167694. 10.1016/j.bbadis.2025.167694 39864669

[phy270385-bib-0022] Nadler, S. F. , Weingand, K. , & Kruse, R. J. (2004). The physiologic basis and clinical applications of cryotherapy and thermotherapy for the pain practitioner. Pain Physician, 7, 395–399. 16858479

[phy270385-bib-0023] Naito, H. , Powers, S. K. , Demirel, H. A. , Sugiura, T. , Dodd, S. L. , & Aoki, J. (2000). Heat stress attenuates skeletal muscle atrophy in hindlimb‐unweighted rats. Journal of Applied Physiology, 88, 359–363. 10642402 10.1152/jappl.2000.88.1.359

[phy270385-bib-0024] Tupling, A. R. (2004). The sarcoplasmic reticulum in muscle fatigue and disease: Role of the sarco(endo)plasmic reticulum Ca2+‐ATPase. Canadian Journal of Applied Physiology, 29, 308–329. 15199229 10.1139/h04-021

[phy270385-bib-0025] Tupling, A. R. , Gramolini, A. O. , Duhamel, T. A. , Kondo, H. , Asahi, M. , Tsuchiya, S. C. , Borrelli, M. J. , Lepock, J. R. , Otsu, K. , Hori, M. , MacLennan, D. H. , & Green, H. J. (2004). HSP70 binds to the fast‐twitch skeletal muscle sarco(endo)plasmic reticulum Ca2+ ‐ATPase (SERCA1a) and prevents thermal inactivation. The Journal of Biological Chemistry, 279, 52382–52389. 15371420 10.1074/jbc.M409336200

[phy270385-bib-0026] Viner, R. I. , Ferrington, D. A. , Huhmer, A. F. , Bigelow, D. J. , & Schoneich, C. (1996). Accumulation of nitrotyrosine on the SERCA2a isoform of SR Ca‐ATPase of rat skeletal muscle during aging: A peroxynitrite‐mediated process? FEBS Letters, 379, 286–290. 8603707 10.1016/0014-5793(95)01530-2

[phy270385-bib-0027] Viner, R. I. , Krainev, A. G. , Williams, T. D. , Schoneich, C. , & Bigelow, D. J. (1997). Identification of oxidation‐sensitive peptides within the cytoplasmic domain of the sarcoplasmic reticulum Ca2+‐ATPase. Biochemistry, 36, 7706–7716. 9201911 10.1021/bi970058z

[phy270385-bib-0028] Viner, R. I. , Williams, T. D. , & Schoneich, C. (1999). Peroxynitrite modification of protein thiols: Oxidation, nitrosylation, and S‐glutathiolation of functionally important cysteine residue(s) in the sarcoplasmic reticulum Ca‐ATPase. Biochemistry, 38, 12408–12415. 10493809 10.1021/bi9909445

[phy270385-bib-0029] Von Schulze, A. T. , Deng, F. , Fuller, K. N. Z. , Franczak, E. , Miller, J. , Allen, J. , McCoin, C. S. , Shankar, K. , Ding, W. X. , Thyfault, J. P. , & Geiger, P. C. (2021). Heat treatment improves hepatic mitochondrial respiratory efficiency via mitochondrial remodeling. Function (Oxf), 2, zqab001. 33629069 10.1093/function/zqab001 PMC7886620

[phy270385-bib-0030] Wolfe, R. R. (2006). The underappreciated role of muscle in health and disease. The American Journal of Clinical Nutrition, 84, 475–482. 16960159 10.1093/ajcn/84.3.475

[phy270385-bib-0031] Yoshihara, T. , Sugiura, T. , Yamamoto, Y. , Shibaguchi, T. , Kakigi, R. , & Naito, H. (2015). The response of apoptotic and proteolytic systems to repeated heat stress in atrophied rat skeletal muscle. Physiological Reports, 3, e12597. 26508739 10.14814/phy2.12597 PMC4632963

[phy270385-bib-0032] Zhang, X. , Wang, C. , Dou, Q. , Zhang, W. , Yang, Y. , & Xie, X. (2018). Sarcopenia as a predictor of all‐cause mortality among older nursing home residents: A systematic review and meta‐analysis. BMJ Open, 8, e021252. 10.1136/bmjopen-2017-021252 PMC6252774 30420343

[phy270385-bib-0033] Zhou, H. H. , Liao, Y. , Peng, Z. , Liu, F. , Wang, Q. , & Yang, W. (2023). Association of muscle wasting with mortality risk among adults: A systematic review and meta‐analysis of prospective studies. Journal of Cachexia, Sarcopenia and Muscle, 14, 1596–1612. 37209044 10.1002/jcsm.13263 PMC10401550

