# Supplementary material for: Heat therapy preserves myofibre size and SERCA‐mediated Ca2+ uptake in the mouse soleus after tenotomy surgery
Source: Physiol Rep. 2025 May 22;13(10):e70385. doi: 10.14814/phy2.70385 (PMC12099063; doi:10.14814/phy2.70385)
Supplement: Supplementary file 1 — Figure S1. [file PHY2-13-e70385-s001.pdf]

Heat therapy preserves myofibre size and SERCA-mediated Ca^{2+} uptake in the mouse soleus after tenotomy surgery

Michael Barfoot[1,2], Jessica L. Braun[1,2], Phil Wallace[1,2], Bianca M. Marcella[1,2], Ryan W. Baranowski[1,2], Rebecca E. K. MacPherson[3], Stephen Cheung[1,2], Val A. Fajardo*[1,2]

[1] Department of Kinesiology, Brock University, St. Catharines, Ontario, Canada
[2] Centre for Bone and Muscle Health, Brock University, St. Catharines, Ontario, Canada
[3] Department of Health Sciences, Brock University, St. Catharines, Ontario, Canada

Supporting Information

*Corresponding author:

Val A. Fajardo
Associate Professor
Department of Kinesiology
Brock University
Ontario, Canada
vfajardo@brocku.ca

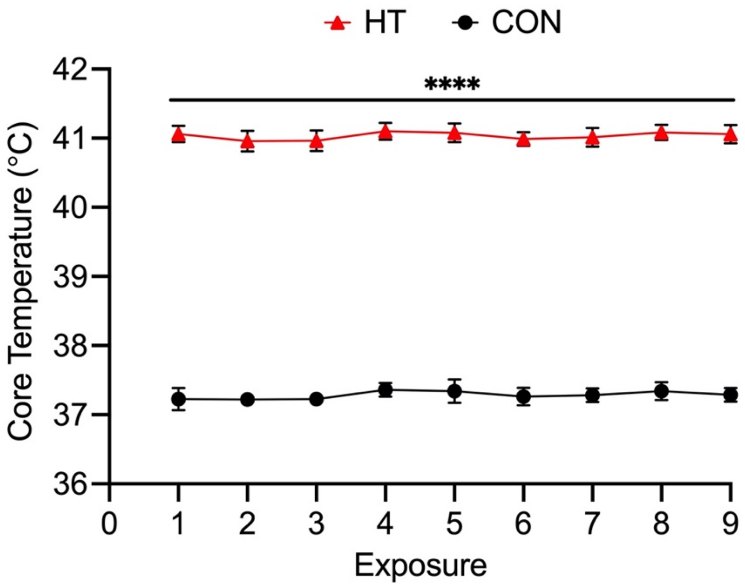

Figure S1. Average core temperature (°C) over the 20-min heat session across the total heating exposures in heat therapy (HT, *n* = 16 males) and control therapy (CON, *n* = 16 males). Data is presented as mean ± SD. Data was analyzed with a 2 (HT vs CON) X 9 (Exposure) repeated measures ANOVA.**** $P<0.0001$ using a t-test at each exposure after adjusting for multiple comparisons with a false discovery rate (Q) = 1%.
